# Zinc Promotes Adipose-Derived Mesenchymal Stem Cell Proliferation and Differentiation towards a Neuronal Fate

**DOI:** 10.1155/2018/5736535

**Published:** 2018-04-18

**Authors:** Mi-Young Moon, Hyun Jung Kim, Bo Young Choi, Min Sohn, Tae Nyoung Chung, Sang Won Suh

**Affiliations:** ^1^Department of Physiology, College of Medicine, Hallym University, Chuncheon, Republic of Korea; ^2^Department of Nursing, Inha University, Incheon, Republic of Korea; ^3^Department of Emergency Medicine, CHA University School of Medicine, Seongnam, Republic of Korea

## Abstract

Zinc is an essential element required for cell division, migration, and proliferation. Under zinc-deficient conditions, proliferation and differentiation of neural progenitors are significantly impaired. Adipose-derived mesenchymal stem cells (AD-MSCs) are multipotent stem cells that can differentiate into neurons. The aim of this study was to evaluate the effect of zinc on AD-MSC proliferation and differentiation. We initially examined the effect of zinc on stem cell proliferation at the undifferentiated stage. AD-MSCs showed high proliferation rates on day 6 in 30 *μ*M and 100 *μ*M of ZnCl_2_. Zinc chelation inhibited AD-MSC proliferation via downregulation of ERK1/2 activity. We then assessed whether zinc was involved in cell migration and neurite outgrowth during differentiation. After three days of neuronal differentiation, TUJ-1-positive cells were observed, implying that AD-MSCs had differentiated into early neuron or neuron-like cells. Neurite outgrowth was increased in the zinc-treated group, while the CaEDTA-treated group showed diminished, shrunken neurites. Furthermore, we showed that zinc promoted neurite outgrowth via the inactivation of RhoA and led to the induction of neuronal gene expression (MAP2 and nestin) in differentiated stem cells. Taken together, zinc promoted AD-MSC proliferation and affected neuronal differentiation, mainly by increasing neurite outgrowth.

## 1. Introduction

Adipose-derived mesenchymal stem cells (AD-MSCs) are multipotent stem cells that have the capacity to differentiate into osteoblasts, chondrocytes, adipocytes, and neuron-like cells [1]. AD-MSCs are superior to bone marrow-derived MSCs in some aspects including ease of culture, rapid growth, and longevity [2]. Moreover, adipose tissue is abundant and easily obtained and can serve as an ideal candidate for cell replacement therapy [3]. Many studies have been conducted to characterize the differentiation potential of AD-MSCs. In particular, the ability to induce neuronal differentiation has elicited great interest in their potential use in the treatment of neurological disease [4–7].

Zinc is an essential element, which is necessary for organ and tissue growth and development [8]. Over 300 enzymes require zinc for their proper function [9], thus zinc is involved in the regulation of various cellular processes including cell division and DNA synthesis [10]. Zinc also affects the growth factor control of cell division, especially in cells regulated by insulin-like growth factor-I (IGF-I) [10] or nerve growth factor (NGF) [11]. Zinc is the second most plentiful transition metal after iron in the central nervous system [12]. Most chelatable zinc is contained in vesicles localized in the presynaptic terminals of the dentate granule cells that form the mossy fiber tracts of the hippocampus [13, 14], a region where neurogenesis and neural migration actively occurs in the adult brain [15, 16]. Additional evidence suggests that zinc may be a key element in neurogenesis and cognitive function and that zinc deficiency may impair neuronal differentiation [17–19]. A recent study also demonstrated that gestational zinc deficiency impaired fetal neuronal progenitor cell proliferation by reducing the ERK1/2 signaling pathway [20].

Despite the significance of zinc in cellular biology, there is little known about the role of zinc in stem cell differentiation and proliferation. Only a handful of studies have been conducted using bone marrow-derived MSCs (BM-MSCs) to address questions regarding zinc and its cellular regulation in stem cells through proteins such as zinc transporters [21, 22]. No studies have addressed the effects of zinc on AD-MSC proliferation, possibly due to the relatively late discovery of AD-MSCs when compared with BM-MSCs. Thus, the present study is aimed at elucidating the potential role of zinc in AD-MSC proliferation and neurogenic differentiation.

## 2. Materials and Methods

### 2.1. Ethics Statement

This study was carried out in strict accordance with the recommendations detailed in the Guide for the Care and Use of Laboratory Animals of the National Institutes of Health. Animal studies were approved by the Committee on Animal Use for Research and Education at Hallym University (protocol number Hallym 2012-28). The institutional review board of CHA Bundang Medical Center, CHA University approved the entire process related to the use of human AD-MSCs used in the current study (IRB approval number BD2012-079D).

### 2.2. Materials

Fetal bovine serum (FBS), DMEM, alpha MEM, gentamycin, phosphate-buffered saline (PBS), and 0.25% Trypsin EDTA were purchased from Invitrogen (CA, USA). 2-Mercaptoethanol (BME), zinc chloride (ZnCl_2_), clioquinol (CQ), calcium disodium ethylene diamine tetraacetate (CaEDTA), Triton X-100, dimethylsulfoxide (DMSO), butylated hydroxyanisole (BHA), anti-mouse IgG-HRP, and anti-rabbit IgG-HRP were purchased from Sigma (MO, USA). Anti-TUJ-1 and Alexa 488 were purchased from Abcam (MA, USA). Anti-phospho-ERK1/2, Anti-ERK1/2, and skim milk were purchased from Santa Cruz (CA, USA). Alexa Fluor 647 anti-human CD90 and FITC anti-human CD105 were purchased from BioLegend (CA, USA). PVDF membrane and ECL were purchased from Bio-Rad (CA, USA). PMSF and protease inhibitor were purchased from Roche (Mannheim, Germany). M-PER mammalian protein extraction reagent was purchased from Thermo Fisher Scientific (IL, USA). CCK-8 was purchased from Dojindo Laboratories (Kumamoto, Japan). FGF-basic (bFGF) was purchased from PeproTech (CA, USA). Bovine serum albumin (BSA) was purchased from BioShop (CA, USA).

### 2.3. Preparation and Culture of AD-MSCs

Human AD-MSCs were isolated as described previously [23]. Adipose tissue was obtained with written informed consent from healthy female donors undergoing elective liposuction procedures at the Department of Plastic Surgery, CHA Bundang Medical Center, CHA University, Gyeonggi-Do, Korea. The collected tissue was mixed with the same volume of PBS with 2% gentamicin and was enzymatically digested with a mixture of trypsin, DNase I, and collagenase I at 37°C for 60 minutes under shaking conditions. The digested tissue was centrifuged at 1500 rpm for 5 minutes and resuspended in saline twice. The cell pellet was filtered through a 100 *μ*m pore-size filter and centrifuged once more to separate the adipose tissue-derived stem cells from the surrounding tissue. Next, 2 × 10^5^ isolated cells were expanded with 15 mL of the culture medium (*α*-minimal essential medium with 10% fetal bovine serum, 1% penicillin/streptomycin) in a T75 flask and cultured in a humidified atmosphere with 5% CO_2_ in an incubator at 37°C. The culture medium was changed every 3 days, and the cells were passaged at 70–80% confluence. Fluorescence-activated cell sorting analysis was used to identify the phenotype of the cells. The expression of CD44, CD73, CD90, CD105, and human leukocyte antigen (HLA)-ABC and the lack of CD45, CD34, CD31, and HLA-DR were checked to confirm the MSC identity. For all experiments in our study, we used cells at passage numbers 5–9.

### 2.4. Induction of Neuronal Differentiation

The procedures were adopted from Woodbury et al. [24]. In brief, AD-MSCs at 1.15 × 10^3^ cells/cm^2^ were seeded in DMEM with 20% FBS one day before preinduction. Twenty-four hours prior to neuronal induction, the medium was replaced with preinduction medium containing DMEM, 20% FBS, and 1 mM 2-mercaptoethanol. To induce neuronal differentiation, the preinduction medium was eliminated, and the cells were rinsed with PBS and changed to a neuronal induction medium composed of DMEM, 1% dimethylsulfoxide (DMSO), and 100 *μ*Μ butylated hydroxyanisole (BHA).

### 2.5. Immunofluorescence

The degree of neurogenic differentiation of AD-MSCs was evaluated by immunocytochemistry. Cells were washed with PBS three times and fixed in 4% paraformaldehyde for 10 min at room temperature. Cells were washed with PBS twice and incubated for 10 min with PBS containing 0.2% Triton X-100. After 10 min, cells were washed in PBS twice and incubated in 5% BSA in PBS for 20 min. Cells were washed in PBS containing 0.1% Triton X-100 three times and incubated overnight with anti-TUJ-1 in the diluted antibodies (1 : 1000) with 5% BSA in PBS. Cells were incubated with DAPI for staining of nuclei (DAPI; Sigma-Aldrich). Cells were washed with TBS containing 0.1% Triton X-100 for 5 min three times. Cells were then incubated with the following secondary antibodies: Alexa 488 (TUJ-1) in TBS for 1 h and washed three times with TBS containing 0.1% Triton X-100 for 5 min each in the dark. Images were taken on a Carl Zeiss LSM710 confocal microscope (Carl Zeiss, Jena, Germany) and analyzed using Zen microscopy software.

### 2.6. Western Blotting

Cells were harvested and rinsed twice with cold PBS, and 100 *μ*L of RIPA lysis buffer (150 mM Tris-HCl, pH 7.4, 150 mM NaCl, 1% Nonidet P-40, 0.25% NaN_3_, 1 mM EDTA, 1 mM PMSF, 1 *μ*g/mL aprotinin, 1 *μ*g/mL leupeptin, 1 *μ*g/mL pepstatin, 1 mM sodium orthovanadate, and 1 mM NaF) was added to each well. The protein-containing samples were resolved by electrophoresis on a 10% SDS-PAGE gel. The separated proteins were transferred to a PVDF membrane at 80 V for 90 min. The membranes were blocked in a 5% skim milk/TBS-Tween 20 solution for 1 h at room temperature, followed by the application of the monoclonal antibody specific for p-ERK1/2 and ERK1/2 at 1 : 1000 in 5% skim milk/TBS-Tween 20. After incubating overnight with the primary antibodies at 4°C, the secondary antibody, anti-rabbit IgG-HRP at 1 : 5000 in 5% BSA/TBS-Tween 20, was applied for 1 h at room temperature. The membrane was washed three times for 5 min after each antibody application. The proteins on the PVDF membrane were detected with an ECL detection system. All experiments were performed three times independently, and each experiment was carried out in triplicate.

### 2.7. Pull-Down Assay for Activated RhoA

AD-MSCs were harvested and washed once with cold PBS, then lysed in lysis buffer (100 mM NaCl, 100 mM MgCl_2_, 20 mM HEPES, pH 7.5, 10% glycerol, 0.5% Nonidet P-40, 0.2% deoxycholate, 1 *μ*g/mL aprotinin, 1 *μ*g/mL leupeptin, and 1 mM PMSF). The lysates were centrifuged at 15,000*g* for 30 min at 4°C. Supernatants were normalized for protein concentration by the Bradford assay, incubated with glutathione s-transferase- (GST-) Rhotekin-Rho-binding domain (RBD) for GTP-RhoA measurement, and GSH-Sepharose 4B beads were rinsed three times with lysis buffer. Bound proteins were eluted by boiling twice in a Laemmli sample buffer. Samples were electrophoresed and measured by Western blot assay with anti-RhoA antibodies. All experiments were performed three times independently, and each experiment was carried out in triplicate.

### 2.8. Proliferation Assay

AD-MSCs were plated onto 96-well culture plates at a density of 100 cells/well and then treated with ZnCl_2_ for 6 days prior to quantification. The medium was replaced every day. Cell proliferation was measured using a Dojindo cell counting kit-8 (CCK-8) (Dojindo Molecular Technologies, Rockville, MD), following the manufacturer's instructions. The culture medium was removed, and 100 *μ*L of fresh medium containing 10 *μ*L CCK-8 was added to each well. The cells were then incubated at 37°C for 1 h and analyzed at 450 nm by a Spectra Max 250 microplate reader (Molecular Devices, Sunnyvale, CA, USA). Control cells (without the addition of ZnCl_2_) had their medium changed every day in the same way as the ZnCl_2_-treated cells.

### 2.9. Real-Time Quantitative Reverse Transcription (RT) PCR

Total AD-MSC RNA was extracted with Isol-RNA lysis reagent (5 Prime Inc., MD, USA). Reverse transcription of the mRNA and polymerase chain reactions (PCR) were performed using the Superscript II system (Invitrogen, Carlsbad, CA) in accordance with the manufacturer's instructions. Primers were as follows: nestin, forward, 5′-TCAACAGCGACGGAGGTCTCTAGGG-3′, and reverse, 5′-CCGCAGACTTCAGTGATTCTAGGAT-3′; MAP2, forward, 5′-CGAAGCGCCAATGGATTCC-3′, and reverse, 5′-TGAACTATCCTTGCAGACAC-3′; and glyceraldehyde-3-phosphate dehydrogenase (GAPDH), which was used as an internal control, forward, 5′-AACGGATTTGGCCGTATT-3′, and reverse, 5′-ACTGTGGTCATGAGCCCTT-3′ (Kodama, 2006, 16519739). Real-time quantitative RT-PCR was measured using a SYBR mixture (Enzynomics, Korea). All experiments were performed three times independently, and each experiment was carried out in triplicate.

### 2.10. FACS Analysis

FACS analysis was performed with freshly isolated CD90^+^ and CD105^+^ cells from AD-MSCs. At least 7 × 10^6^ cells (in 100 *μ*L PBS/0.5% BSA) were incubated with fluorescence-labeled monoclonal antibodies (1/20 diluted, 4°C, 1 hour). For staining, anti-human CD90 Alexa Fluor 647 and anti-human CD105 FITC were used. The labeled cells were washed twice and measured by flow cytometry by use of the FACSCanto II (Becton Dickinson, NJ, USA) flow cytometer and the WinMDI 2.9 software (Scripps Institute). All experiments were performed three times independently, and each experiment was carried out in triplicate.

### 2.11. Measurement of Neurite Length

Neurite outgrowth was assayed in neuron-like cells. After 3 days from initial plating, the medium was changed to promote neuronal differentiation and the length of neurite outgrowth was measured 3 days later. Using a 20x objective, a sufficient number of fields were acquired for the analysis of at least 70 cells per well. Neurite length was evaluated by using ImageJ (NCBI, MD). The longest process was measured and averaged. All experiments were performed three times independently, and each experiment was carried out in triplicate.

### 2.12. Statistical Analysis

Data are presented as means ± SD. The *t*-test was used to compare groups using the GraphPad Prism program (GraphPad Software, San Diego, CA).

## 3. Results

### 3.1. Zinc Increases Proliferation of AD-MSCs

**Figure 1:**
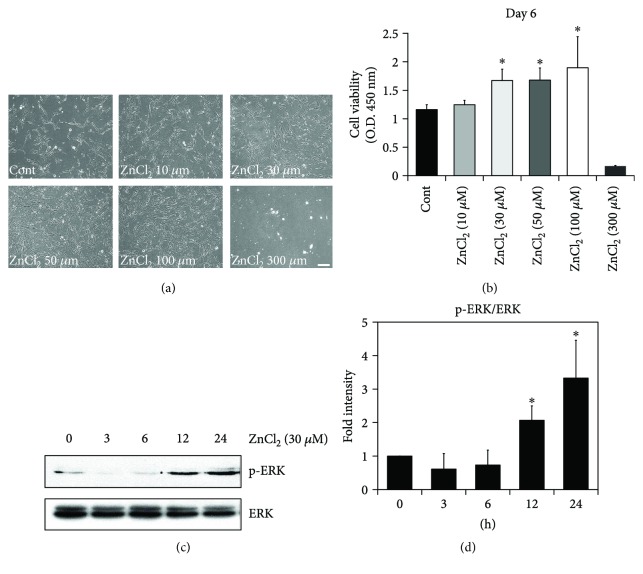
Zinc increases proliferation of AD-MSCs. To evaluate the role of zinc in cellular proliferation, AD-MSCs were cultured in 6-well plates at 1 × 10^4^ cells per well with culture media containing zinc at the following concentrations: 0, 10, 30, 50, 100, and 300 *μ*Μ. AD-MSC proliferation was analyzed at day 6. AD-MSC proliferation was increased by zinc supplement. (a) Phase-contrast photomicrographs of AD-MSCs 6 days after the addition of 0, 10, 30, 50, 100, and 300 *μ*M ZnCl_2_. AD-MSC proliferation was significantly increased by zinc supplements in a dose-dependent manner, except at 300 *μ*M. Scale bar, 100 *μ*m. (b) Effect of zinc on AD-MSC proliferation was evaluated using the CCK-8 assay each day for 6 days. AD-MSC proliferation was higher in 100 *μ*M of zinc when compared with controls. Data are representative of ten independent experiments (42%, 43%, and 75%, resp., ^∗^*p* < 0.05 versus untreated with ZnCl_2_). (c) AD-MSCs were incubated with 30 *μ*Μ ZnCl_2_ for 3, 6, 12, and 24 h and then lysed. Western blotting was performed with anti-phospho-ERK1/2 and anti-ERK1/2 antibodies. (d) Data are expressed as means ± SD of three independent experiments (^∗^*p* < 0.05 versus untreated with ZnCl_2_).

It has been reported that zinc deficiency restricts cellular proliferation and growth [18]. Likewise, the replenishment of zinc restores the progenitor pool by preventing the degeneration of newly born cells and also by supporting de novo synthesis. Based on the above study, we hypothesized that zinc may promote AD-MSC proliferation. First, to examine whether zinc supplementation affected AD-MSC proliferation, AD-MSCs were cultured in various concentrations of ZnCl_2_-containing media for 6 days. Phase-contrast photomicrographs showed that AD-MSC growth increased in a dose-dependent manner with zinc concentration. Cell viability of AD-MSCs, quantified with the CCK-8 assay, increased moderately in 30 and 50 *μ*Μ and prominently in 100 *μ*Μ of ZnCl_2_ when compared to the controls (42%, 43%, and 75%, resp., *p* < 0.001). However, at 300 *μ*Μ of ZnCl_2_, cytotoxicity was observed (Figures 1(a) and 1(b)). Based on this result, subsequent experiments were conducted with 30 *μ*M of ZnCl_2_. Previous studies have suggested that self-renewal and proliferation of stem cells is dependent on the activation of the ERK pathway [25, 26]. Thus, we tested whether ERK activation was required for AD-MSC proliferation. Upon the addition of ZnCl_2_ to the cell culture medium, we observed an increase in the phosphorylated form of ERK 1/2 in AD-MSCs (Figures 1(c) and 1(d)). These data indicate that the ability of ZnCl_2_ to increase AD-MSC proliferation may be mediated in part by ERK activation.

### 3.2. Zinc Chelation Inhibits Cellular Growth through Downregulation of ERK1/2 Activity

**Figure 2:**
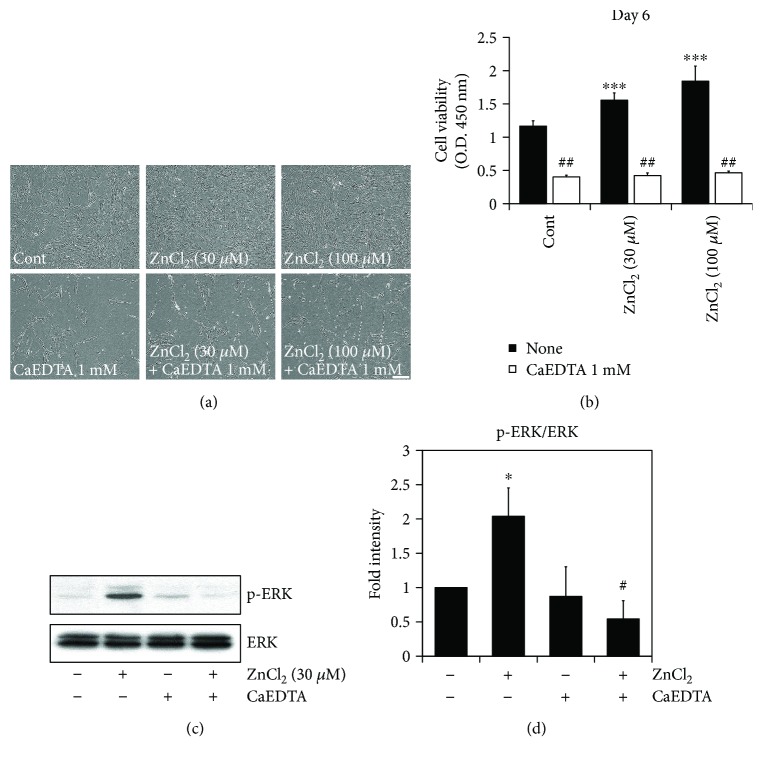
Zinc chelation inhibits cellular growth through downregulation of ERK1/2 activity. To investigate whether extracellular zinc chelation affected AD-MSC survival, cultured AD-MSCs were treated with 1 mM CaEDTA in the culture medium. AD-MSC survival was inhibited by the addition of zinc chelators. (a) Phase-contrast photomicrographs of AD-MSCs 6 days after the addition of zinc chelators. Scale bar, 100 *μ*m. (b) The effect of zinc chelators on AD-MSC survival was evaluated using the CCK-8 assay for 6 days. AD-MSCs were pretreated with or without 1 mM CaEDTA and then incubated with ZnCl_2_ (30 *μ*M and 100 *μ*M) for 6 days (^∗∗∗^*p* < 0.0001 versus untreated with ZnCl_2_; ^##^*p* < 0.001 versus untreated with CaEDTA). (c) To determine whether chelation of zinc affected the activity of ERK, AD-MSCs were treated with or without CaEDTA and then incubated with ZnCl_2_ for 24 h. Western blotting using phospho-ERK1/2 and ERK1/2 antibodies was performed. (d) These data represent the mean ± SD from three independent experiments (^∗^*p* < 0.05 versus untreated with ZnCl_2_; ^#^*p* < 0.05 versus treated with ZnCl_2_).

To verify that the increased cell survival was due to zinc, the extracellular zinc chelator CaEDTA was added to the zinc-containing media. Phase-contrast photomicrographs demonstrated that zinc chelation showed less AD-MSC (Figures 2(a) and 2(b)) and resulted in the restriction of AD-MSC survival within a constant range (from 0.40 to 0.49) regardless of the initial dose of ZnCl_2_ (Figures 2(a) and 2(b)).

We also conducted Western blotting with antibodies against ERK1/2, which is a well-established signaling pathway used to estimate proliferation rates. An ERK1/2 assay was used to address the cellular mechanism of cell survival under either zinc rich or deficient conditions. The present study showed that zinc chelation suppressed the ERK1/2 signaling pathway (Figures 2(c) and 2(d)).

### 3.3. Zinc Promotes the Neuronal Differentiation of AD-MSC

**Figure 3:**
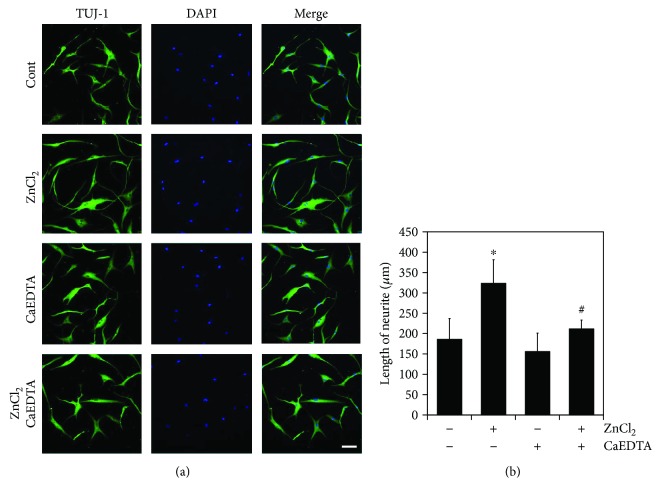
Zinc promotes neuronal differentiation of AD-MSCs. Zinc affects the differentiation of AD-MSCs into neuron-like cells. AD-MSCs were cultured in neuronal differentiation medium containing either 0 or 10 *μ*M ZnCl_2_ with or without CaEDTA. TUJ-1 expression was assessed through immunocytochemistry, and neurite length assay was performed as described above. The results showed that zinc promoted neurite outgrowth and expression of the neuronal marker TUJ-1. (a) Immunocytochemistry for TUJ-1 in treated AD-MSCs. Scale bar, 100 *μ*m. (b) Neurite length in treated AD-MSCs. Experiments were performed on at least 70 cells and represent the mean ± SD from three independent experiment (^∗^*p* < 0.05 versus untreated with ZnCl_2_; ^#^*p* < 0.05 versus treated with ZnCl_2_).

To test our hypothesis that zinc was involved in cell migration and neurite outgrowth by modulating cytoskeletal dynamics during differentiation, AD-MSCs were cultured in neuronal differentiation media using a modified version of the Woodbury protocol under various concentrations of zinc. AD-MSC morphology began to change 1 h after exposure to induction agents with the extension of processes. After 3 days, the cultured cells assumed a more sharpness and shifted morphologically into neuron-like cells. Immunocytochemistry revealed that these cells expressed the immature neuronal marker, TUJ-1 (Figure 3(a)).

Neurite length was measured from photographed fluorescent images at different zinc concentrations. As expected, zinc increased neurite length in a dose-dependent manner (Figure 3(b)). However, excessive zinc concentrations induced cellular degeneration during the neurogenic induction process (data not shown).

### 3.4. Zinc Promotes Neuronal Differentiation of AD-MSC via Downregulation of RhoA

**Figure 4:**
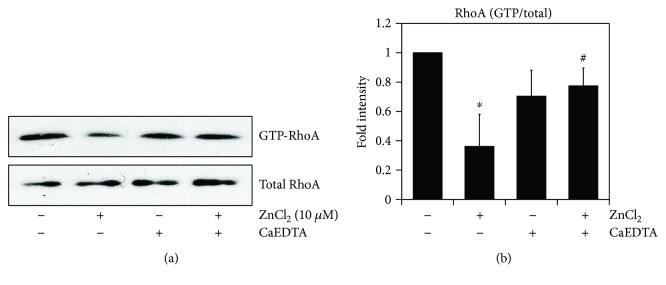
Zinc promotes neuronal differentiation of AD-MSCs via downregulation of RhoA. Inactivation of RhoA is involved in neurite outgrowth of differentiated AD-MSCs. (a) Differentiated AD-MSCs were treated with or without CaEDTA and then incubated with ZnCl_2_. GTP-RhoA levels were measured by pull-down assays with GST-RBD fusion protein and Sepharose beads. RhoA expression was determined with anti-RhoA antibody and Western blotting. (b) Proteins used in Western blotting were quantified by densitometry using ImageJ, and the relative amounts of the GTP-bound form in each sample are shown. The ratio of GTP-bound form/total protein in untreated cells was set as 1, and the data are means ± SD of at least three independent experiments (^∗^*p* < 0.05 versus untreated with ZnCl_2_; ^#^*p* < 0.05 versus treated with ZnCl_2_).

During the initial stage of differentiation, V14RhoA (activated form) inhibits the initiation of neuronal differentiation, whereas the inactivated form of RhoA appears necessary for neurite outgrowth [27]. RhoA inactivation is required for neurite outgrowth in PC12 cells, and cAMP and phosphorylated RhoA (S188D) have been shown to induce neurite outgrowth [28]. To test if the neuronal differentiation of zinc-treated AD-MSCs required RhoA activity, AD-MSCs were cultured in neuronal differentiation media and treated with 10 *μ*Μ ZnCl_2_. Zinc reduced GTP-RhoA and then chelation of zinc using CaEDTA was sufficient to restore RhoA activity (Figures 4(a) and 4(b)). We show here that inactivated RhoA promoted neurite outgrowth in AD-MSC zinc treated.

### 3.5. Zinc Increases Neuronal Gene Expression in Differentiated AD-MSCs

**Figure 5:**
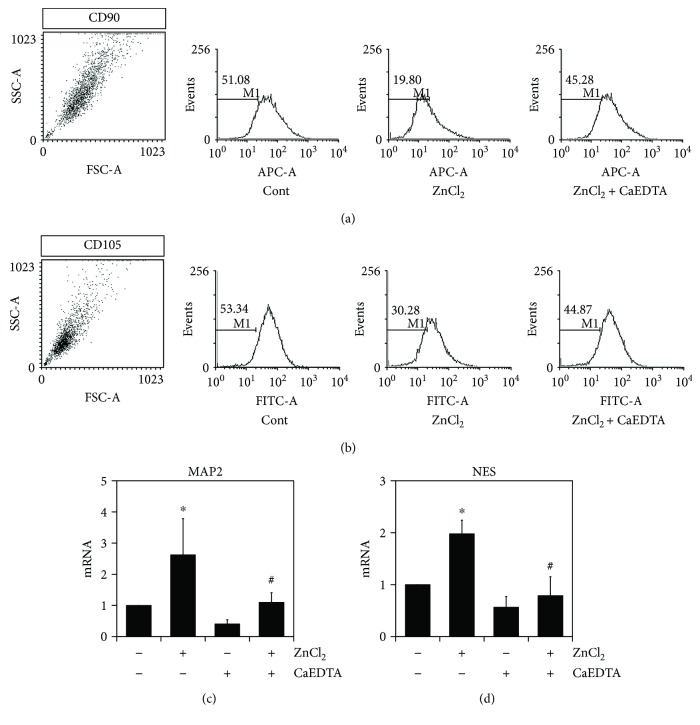
Zinc induces neuronal gene expression in differentiated AD-MSCs. Effects of zinc on the expression of NES and MAP2 during neuronal differentiation in AD-MSCs. (a, b) To investigate whether zinc played a role in the expression of the surface protein molecules CD90 and CD105, flow cytometry was performed in differentiated AD-MSCs. AD-MSCs were incubated in differentiation medium with the presence of either 10 *μ*M ZnCl_2_ or CaEDTA for 3 days. Analysis of the expression of CD90 and CD105 was determined by WinMDI software. (c, d) mRNA expression of NES and MAP2 was measured by real-time quantitative RT-PCR. These data represent the mean ± SD from three independent experiments (^∗^*p* < 0.05 versus untreated with ZnCl_2_; ^#^*p* < 0.05 versus treated with ZnCl_2_).

We hypothesized that zinc could be synergistically induced to differentiate stem cells to neurons. AD-MSCs express CD29, CD44, CD90, and CD105 in the undifferentiated state [29]. First, we investigated whether exposure to zinc could decrease the expression of cell surface markers in AD-MSCs. Differentiated AD-MSCs were analyzed by FACS for the expression of CD90 and CD105. AD-MSCs cultured in neuronal differentiation media with ZnCl_2_ showed a reduced expression of CD90 and CD105, which was restored by CaEDTA treatment (Figures 5(a) and 5(b)). ZnCl_2_ also promoted the expression of neuronal markers such as MAP 2 (microtubule-associated protein 2) and NES (nestin) (Figures 5 and 5). Taken together, these results showed that the AD-MSCs rapidly and efficiently differentiated into neuron-like cells in the presence of zinc.

## 4. Discussion

In this study, we showed that zinc supplementation increased AD-MSC proliferation and neurite outgrowth. These findings confirmed our hypothesis that moderate doses (30–100 *μ*Μ) of zinc promoted proliferation through ERK1/2 activation. These results are in agreement with previous studies by Vega-Robledo et al. [30], demonstrating that zinc acts as a sort of double-edged sword with respect to cell viability in various cell types. Even though the concentration-dependent effects on cell viability were different for each cell type and passage number, the evidence outlined above further confirmed that zinc is a pivotal element in cell growth and proliferation.

Time-dependent assays revealed that zinc maximally accelerated cell proliferation after 6 days following the beginning of treatment. Up to three days, the growth rate was relatively slow among all three groups, as it was between the incubation periods (data not shown). This delayed response suggests that zinc may act via chronic cell proliferation-regulating molecules. Indeed, zinc has long been recognized as a crucial modulator of the function of a diverse number of enzymes, hormones, and transcription factors, making our hypothesis more persuasive [10].

It has been broadly accepted that zinc deficiency causes growth retardation and developmental arrest. Zinc deprivation studies have also suggested that the reduction of zinc liberation retards progenitor cell expansion in multiple tissue types. As shown in Figure 2, zinc chelation restricted AD-MSC proliferation in accordance with results presented in previous studies [31].

The cellular mechanisms that underlie the cell biological functions of zinc are not yet fully understood. However, several potential molecules involved in the regulation and action of zinc have been identified such as ERK1/2, BDNF, and JNK [32–34]. This study evaluated ERK1/2 due to its known role in proliferation. Western blotting demonstrated a diminished expression of ERK1/2 in the zinc chelation groups, especially, in CaEDTA. Even though CaEDTA and CQ are known to be extracellular zinc chelators, they work through distinct cellular mechanisms [19].

Other than the observation that zinc is associated with cellular proliferation, zinc is known to affect neurogenesis in the hippocampus [19, 35–37]. Additionally, converging lines of evidence support the idea that zinc promotes neuronal differentiation not only in neuronal progenitor cells, but also in other types of stem cells such as bone marrow mesenchymal stem cells [38, 39]. Thus, we hypothesized that zinc's ability to promote the process of hippocampal neurogenesis may be attributed to its ability to drive neurogenic differentiation of stem cells, as seen here in AD-MSCs. The observed morphological changes and immunocytochemistry results clearly demonstrated the possibility of neural induction. However, Foudah et al. [40] reported that some undifferentiated stem cells also expressed similar “neuronal” markers, making our results difficult to definitively interpret. Therefore, a deeper understanding of the process of differentiation than what is currently available will be required to fully evaluate the criteria best able to determine the true “fate” of differentiated cells. However, despite our inability to definitively confirm differentiation into “true” neurons by immunocytochemistry, zinc had a clear, positive effect on neurite outgrowth, indicating its role in this process.

Taken together, the present study demonstrates that zinc enhanced AD-MSC proliferation and promoted differentiation towards a neuronal fate in these cells. Determining the mechanism of action for these effects as well as further elaboration of the cell types involved in zinc-mediated cellular biology warrants further investigation.
